# Subjective experiences during dexmedetomidine- or propofol-induced unresponsiveness and non-rapid eye movement sleep in healthy male subjects

**DOI:** 10.1016/j.bja.2023.04.026

**Published:** 2023-05-31

**Authors:** Katja Valli, Linda Radek, Roosa E. Kallionpää, Annalotta Scheinin, Jaakko Långsjö, Kaike Kaisti, Oskari Kantonen, Jarno Korhonen, Tero Vahlberg, Antti Revonsuo, Harry Scheinin

**Affiliations:** 1 Department of Psychology and Speech-Language Pathology, Turku Brain and Mind Center, University of Turku, Turku, Finland; 2 Department of Perioperative Services, Intensive Care and Pain Medicine, Turku University Hospital, Turku, Finland; 3 Department of Cognitive Neuroscience and Philosophy, School of Bioscience, University of Skövde, Skövde, Sweden; 4 Turku PET Centre, University of Turku and Turku University Hospital, Turku, Finland; 5 Department of Intensive Care, Tampere University Hospital, Tampere, Finland; 6 Department of Anesthesiology and Intensive Care, Oulu University Hospital, Oulu, Finland; 7 Institute of Clinical Medicine, Biostatistics, University of Turku and Turku University Hospital, Turku, Finland; 8 Institute of Biomedicine and Unit of Clinical Pharmacology, University of Turku and Turku University Hospital, Turku, Finland

**Keywords:** anaesthesia, awareness, consciousness, dexmedetomidine, dreaming, propofol, sleep, unresponsiveness

## Abstract

**Background:**

Anaesthetic-induced unresponsiveness and non-rapid eye movement (NREM) sleep share common neural pathways and neurophysiological features. We hypothesised that these states bear resemblance also at the experiential level.

**Methods:**

We compared, in a within-subject design, the prevalence and content of experiences in reports obtained after anaesthetic-induced unresponsiveness and NREM sleep. Healthy males (*N*=39) received dexmedetomidine (*n*=20) or propofol (*n*=19) in stepwise doses to induce unresponsiveness. Those rousable were interviewed and left unstimulated, and the procedure was repeated. Finally, the anaesthetic dose was increased 50%, and the participants were interviewed after recovery. The same participants (*N*=37) were also later interviewed after NREM sleep awakenings.

**Results:**

Most subjects were rousable, with no difference between anaesthetic agents (*P*=0.480). Lower drug plasma concentrations were associated with being rousable for both dexmedetomidine (*P*=0.007) and propofol (*P*=0.002) but not with recall of experiences in either drug group (dexmedetomidine: *P*=0.543; propofol: *P*=0.460). Of the 76 and 73 interviews performed after anaesthetic-induced unresponsiveness and NREM sleep, 69.7% and 64.4% included experiences, respectively. Recall did not differ between anaesthetic-induced unresponsiveness and NREM sleep (*P*=0.581), or between dexmedetomidine and propofol in any of the three awakening rounds (*P*>0.05). Disconnected dream-like experiences (62.3% *vs* 51.1%; *P*=0.418) and memory incorporation of the research setting (88.7% *vs* 78.7%; *P*=0.204) were equally often present in anaesthesia and sleep interviews, respectively, whereas awareness, signifying connected consciousness, was rarely reported in either state.

**Conclusions:**

Anaesthetic-induced unresponsiveness and NREM sleep are characterised by disconnected conscious experiences with corresponding recall frequencies and content.

**Clinical trial registration:**

Clinical trial registration. This study was part of a larger study registered at ClinicalTrials.gov (NCT01889004).

**Editor's key points**

• Anaesthetic-induced unresponsiveness and non-rapid eye movement (NREM) sleep share common neurophysiological features which might be reflected also at the experiential level.
• This study compared the prevalence and content of experiences in reports obtained from healthy male volunteers after dexmedetomidine- or propofol-induced unresponsiveness and NREM sleep.
• Disconnected dream-like experiences and experiences related to research setting were often present in both anaesthesia and sleep interviews, whilst awareness signifying connected consciousness was rarely reported for either.
• Anaesthetic-induced unresponsiveness and NREM sleep are both characterized by disconnected conscious experiences with similar recall frequencies and content.

Anaesthetic-induced unresponsiveness and non-rapid eye movement (NREM) sleep share similarities on mechanistic, neurophysiological, and subjective levels. Relevant for the current study, both the α_2_-adrenoreceptor agonist dexmedetomidine and γ-aminobutyric acid A agonist propofol activate endogenous sleep-promoting pathways.^1^, ^2^, ^3^, ^4^, ^5^, ^6^ Correspondingly, positron emission tomography (PET) studies have revealed that these anaesthetics and NREM sleep cause a global reduction of cerebral blood flow, with the largest regional reductions in the thalamus and brainstem.^7^, ^8^, ^9^ Notably, emergence from dexmedetomidine- and propofol-induced unresponsiveness, and awakening from N2 sleep involve activation of these same areas.^8^^,^^10^^,^^11^ On the neurophysiological level, general anaesthesia and NREM sleep generate similar, although not identical, changes in canonical EEG frequency bands, most notably an increase in slow wave activity and a decrease in high-frequency activity.^12^, ^13^, ^14^, ^15^, ^16^ Dexmedetomidine induces a state neurophysiologically closest to NREM sleep: dexmedetomidine sedation shows spindle activity typical for N2 sleep, and deeper, unresponsive dexmedetomidine sedation produces strong delta activity, akin to N3 sleep.^14^^,^^17^, ^18^, ^19^

At the subjective level, anaesthetic-induced unresponsiveness and NREM sleep are associated with reports of disconnected conscious experiences, that is, both states seem to support purely internally generated experiences that are not induced by external sensory stimuli. In interviews performed immediately after recovery from general anaesthesia conducted with a broad range of agents (excluding ketamine), 21.7–27.7% of patients indicate remembering dreams.^20^, ^21^, ^22^ Relatively comparable recall rates to surgical anaesthesia, between 11.8% and 39.8%, have been observed upon emergence after clinical sedation utilising propofol.^23^, ^24^, ^25^, ^26^, ^27^ An even higher recall rate of different types of disconnected experiences has been observed in experimental awakenings from dexmedetomidine-induced (58.5–89.8%) or propofol-induced (31.4–73.5%) sedation and unresponsiveness.^28^, ^29^, ^30^, ^31^ These experiences comprise dreaming but often also include references to the research setting.

In systematic sleep laboratory awakenings from NREM sleep, dream recall averages around 43.0% but has varied significantly between studies depending on how dreaming is defined and the timing of the awakenings.^32^ Notably, in those sleep studies where the definition of dreaming has been most comparable with the experimental anaesthesia studies, dream recall rates in NREM sleep serial awakenings have ranged from 30.7% to 41.6%.^15^^,^^33^ Qualitatively, the experiences during both anaesthetic-induced unresponsiveness and early-night NREM sleep are most often composed of brief, pleasant, and visual, auditory, or non-perceptual (‘thought-like’) content that lacks temporal progression and complexity.^22^^,^^25^^,^^28^^,^^30^^,^^33^ Notably, reporting dreams after emergence from surgical anaesthesia has been found to correlate with higher rates of spontaneous dream recall in the home setting.^21^ This suggests that individual trait differences might partly explain between-subject variation in remembering disconnected experiences or relate to specific types of recalled content.

Given the many similarities between anaesthetic-induced unresponsiveness and NREM sleep, we investigated the prevalence and content of subjective experiences in interviews obtained after arousals from dexmedetomidine-induced or propofol-induced unresponsiveness and NREM sleep in the same participants. Direct comparison between the states in a within-subject design was used to account for individual trait differences in recall. We hypothesised that anaesthetic-induced unresponsiveness and NREM sleep are characterised by highly similar rates of recall of subjective experiences and that the contents of the experiences are not systematically different in most respects.

## Methods

This study was part of a larger research project registered in ClinicalTrials.gov (NCT01889004). It was approved by the Ethics Committee of the Hospital District of Southwest Finland and the Finnish Medicines Agency. Written informed consent was acquired from all participants according to the Declaration of Helsinki. PET results have been reported from the same experiments.^8^

### Participants

This open-label parallel-group study consisted of two separate experiments conducted with the same participants. We recruited 40 non-smoking, 20-30-yr-old right-handed healthy males (ASA physical status 1) with normal hearing. The participants were randomised with a permuted block design to receive either dexmedetomidine or propofol. The sample size was based on our previous experience with similar study designs, and no formal statistical power calculation regarding the required number of participants was conducted before the study.

### Experiments

The recruitment, anaesthetic protocol, and experimental details have been published^8^; only the procedures most relevant to the present study are described here.

#### Experiment 1: the anaesthesia experiment

**Fig 1:**
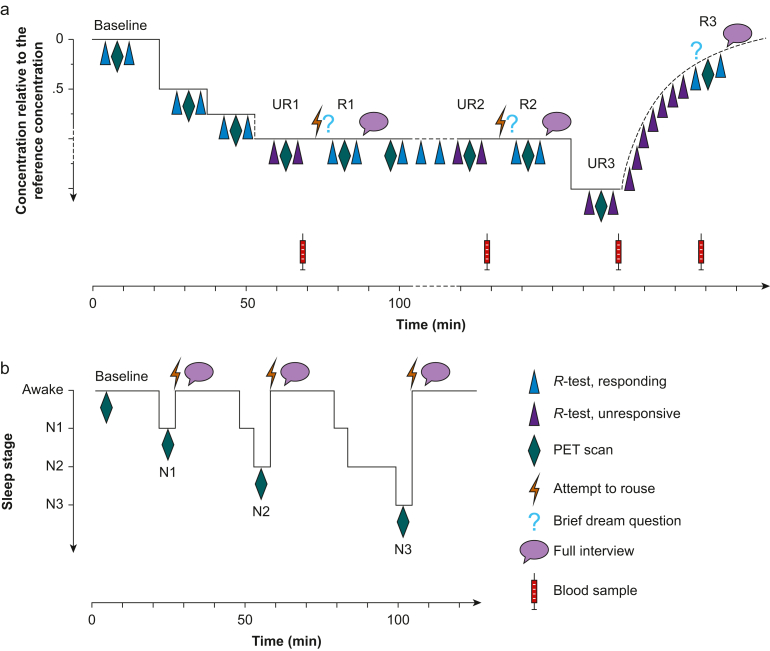
Design of Experiments 1 and 2. (a) Anaesthesia experiment. R1, first regaining of responsiveness; R2, second regaining of responsiveness; R3, recovery to responsiveness; UR1, first period of unresponsiveness; UR2, second period of unresponsiveness; UR3, third period of unresponsiveness with higher drug concentration level. (b) Sleep experiment. N1, NREM sleep stage N1; N2, NREM sleep stage N2; N3, NREM sleep stage N3. NREM, non-rapid eye movement; PET, positron emission tomography.

Participants were randomised to receive either dexmedetomidine (*n*=20; Dexdor® 100 μg ml^−1^; Orion Pharma, Espoo, Finland) or propofol (*n*=19; Propofol-®Lipuro 10 mg ml^−1^; B. Braun, Melsungen, Germany; one participant withdrew after randomisation). In Experiment 1 (Fig 1a), participants rested in the PET scanner in a supine position, and a 64-channel EEG was recorded throughout the experiment. After the baseline PET scan, drug administration was commenced using target-controlled infusion (TCI) with 0.5 times the individual reference concentration for loss of responsiveness, defined in a previous dose-finding study.^16^ Then, the concentration was increased to 0.75 times and finally to 1.0 times the reference concentration.

Participant responsiveness was tested using an auditory responsiveness test (*R*-test) presented via headphones before and after each PET scan (Fig 1a). Each *R*-test was composed of 10 unique sentences with a semantically congruous (*n*=5) or incongruous (*n*=5) last word, and the participants were instructed to respond by squeezing the right or left handle of custom-made handles depending on the congruency of the sentence. Unresponsiveness (UR) was defined as the inability to respond to any item in the *R*-test. If unresponsiveness was not achieved with 1.0 × reference concentration, additional ×1.25 increments were administered at approximately 13-min intervals (comprising the stabilisation period, *R*-tests, and PET scan) until the first unresponsiveness (UR1) ensued. The mean targeted doses (SD) for unresponsiveness were 1.50 (0.56) ng ml^−1^ for dexmedetomidine and 1.78 (0.56) μg ml^−1^ for propofol. During UR1, constant-rate TCI was continued, and a PET scan was obtained. After the UR1 PET scan, the participant was roused by calling him by name and then gently nudging his shoulder. If responsiveness (R) was regained, a brief dream question was immediately asked, ‘Did you have a dream?’ with answer options ‘yes’, ‘no’, and ‘not sure’. This was followed by the R1 PET scan and a full semi-structured interview (Table 1) while the TCI was held constant. The time lapse between the brief dream question and the full interview was ∼7.5 min because of the intervening PET scan.

**Table 1:** Interview questions and content analysis scale.

**The interview questions inExperiments 1 and 2.** **Interview questions 1–6 were presented to each participant in the same way, and depending on the answers, further details (A→) were inquired when applicable.**	
1. Did you dream during anaesthesia/sleep? If the participant answered YES, content of the experience was assessed with: A. Describe the dream in as much detail as possible. B. Where were you, and what the environment of the dream was like? What happened in the dream? What did you see? What did you hear? What did you sense and feel? What did you do? Were you alone? Were there other characters in the dream? What did they do? Did anything else happen? C. Did you have the dream just before the awakening? D. Did you experience feelings/emotions in your dream? What kinds of feelings or emotions did you experience? What dream situation were the emotions related to? Was the dream pleasant or unpleasant? If the participant answered NO, recall certainty was assessed: A. Are you certain you did not dream? B. Do you feel you might have been dreaming but forgot what the dream was about?	
2. Did you experience anything related to this room or situation during anaesthesia/sleep? If the participant answered YES: A. Describe the experience in as much detail as possible. B. What happened? Who were involved? What was the environment like? Do you recall any additional details? If the participant answered NO, the next question was presented.	
3. Did you hear anything during anaesthesia/sleep? If the participant answered YES: A. Describe what you heard in as much detail as possible. B. Can you describe the sounds you heard? Can you describe the speaker's gender? Can you describe the tone or content of what was said? If the participant answered NO, the next question was presented.	
4. Did you sense anything during anaesthesia/sleep? If the participant answered YES: A. Describe what you sensed in as much detail as possible. B. Was the sensation unpleasant or painful? What was the pain like? What part of your body was affected? If the participant answered NO, the next question was presented.	
5. Do you remember anything else from during anaesthesia/sleep that you have not already mentioned?	
6. What is the last thing you remember before falling asleep? What is the first thing you remember after awakening? (These questions were asked after UR3 in Experiment 1 and after each awakening in Experiment 2.)	
**The content analysis scale for the classification of the interview reports.** **All interview transcripts were coded by two independent raters, and in case of disagreement, the content of the report was discussed until an agreement was reached or the final decision was made by a third judge.Inter-rateragreement in all stages of content analysis was substantial in both experiments, ranging from 86.8% to 98.5% between different classifications (*****Ҡ*****-values ranging from 0.736 to 0.972; all*****P*****-values <0.001).**	
**Stage 1**. All interviews were coded for recall of experiences	
No recall	The participant regained responsiveness but was adamant he did not experience anything during unresponsiveness
White report	The participant reported having a strong impression that he had had experiences during unresponsiveness but could not recall any explicit content
Content report	The participant reported having had experiences that have most evidently taken place during the period of unresponsiveness
**Stage 2**. Content reports were further coded to include disconnected and/or connected content	
Disconnected experiences	
Dreaming	Purely internally generated hallucinatory content that was not directly related to or did not originate from the research environment
Memory incorporation	Experiences that realistically or in distorted form depicted objects, persons, events, or sensations/feelings related to the research setting and to which the participant had been exposed to before unresponsiveness/sleep
Connected experiences	
Awareness	Externally generated experiences that were related to objects/persons that had been present, or events that had occurred, during the session, but the occurrence of which the participant could not have anticipated, and which thus could not be categorised as memory incorporation
**Stage 3**. Perceptual complexity and dynamics of experience were coded separately for dream-like experiences, memory incorporation, and awareness	
No sensory–perceptual content	Thought-like, non-perceptual content
Static experience	An isolated, fragmentary, and typically unisensory percept or several connected percepts without temporal progressions occurring within or between percepts
Dynamic experience	Complex, connected, and typically multisensory percepts, which are located within a scene, with temporal progression occurring either between percepts within a scene or between scenes
**Stage 4**. Modality of experiences was coded separately for dreaming, memory incorporation, and awareness	
Sensory–perceptual experiences	Visual, auditory, gustatory, olfactory, interoceptive (e.g. hunger, thirst, heartbeat, and breathing), kinaesthetic/vestibular, tactile, or noci- and thermoceptive experiences
Affective states	Positive and negative moods and emotions
Cognition	Thoughts, memories, inner speech, planning, and reflection of content of consciousness
Out-of-body experience	Observing one's body or the research environment from outside one's physical body
Sense of presence	Sensing a presence of another person/being in the room

Participants were then left unstimulated, and if they achieved a second unresponsive (UR2) period with the same drug concentration as in UR1/R1, the procedure was repeated. Finally, the drug concentration was increased by 50% to achieve a third period of unresponsiveness with a higher drug concentration level (UR3). The mean targeted doses for UR3 were 2.38 (1.05) ng ml^−1^ for dexmedetomidine and 2.74 (0.81) μg ml^−1^ for propofol. After the UR3 PET scan, the infusion was terminated, and the participant's name and a request to press the response handles were repeated until he recovered to a responsive state (R3). This was followed by the brief dream question, blood sampling, PET scan, and full interview. In R3, the median time from termination of infusion to full interview was 32.0 min (range: 8.5–61.1) in participants receiving dexmedetomidine and 14.9 min (range: 9.4–30.0) in participants receiving propofol, that is, the R3 interviews were more delayed compared with interviews in R1 and R2.

Blood samples for drug concentration measurements were drawn at baseline, at the end of each drug target infusion step, and when the responsiveness of the participant changed during the steady infusion or after the infusion was terminated. Dexmedetomidine concentrations in plasma were measured by high-performance liquid chromatography (HPLC) with tandem mass spectrometry, and propofol concentrations were measured by HPLC with fluorescence detection.

#### Experiment 2: the sleep experiment

Two additional participants withdrew before Experiment 2 (*N*=37), which was conducted ∼18 weeks after Experiment 1. In Experiment 2 (Fig 1b), the participants were imaged during sleep-deprived wake and NREM sleep stages N1, N2, and N3. They were awakened and interviewed with the same semi-structured interview (Table 1) after each sleep scan. EEG was recorded as in Experiment 1 with the addition of chin muscle tone measurement for sleep staging. To enhance the probability of falling asleep in the scanner, 30 h sleep deprivation was conducted at home before the sleep imaging sessions, and imaging sessions were conducted in the afternoon to utilise the natural increase in circadian sleep drive.^34^ During the deprivation night, vigilance was monitored with the ZEO sleep monitor (ZEO Inc., Boston, MA, USA), and the participants were also provided a tablet computer with pre-programmed alarms every 20–40 min. The participants had to solve a mathematical problem correctly before the alarm, with volume increasing over time, could be silenced. Based on the ZEO data, two participants fell asleep during the sleep deprivation period, sleeping for a total of 26 min and 1 h and 47 min, respectively.

Sleep stages were identified online by an experienced sleep technician according to the American Academy of Sleep Medicine criteria.^35^ When a participant reached the targeted sleep stage, a PET scan was obtained, and the participant was awakened and interviewed. All interviews in Experiment 2 were conducted immediately after awakenings. The total number of scans was limited to five to avoid excessive radiation exposure. Because of individually alternating intervals of sleep stages, it was not always possible to obtain PET scans and interviews in chronological order from N1, N2, and N3, and scans/awakenings from all targeted sleep stages were not achieved with each participant. After data collection was complete, the sleep technician and another expert in sleep stage scoring verified the vigilance states from the offline polysomnography recording, with an inter-rater agreement of 93.1% (*Ҡ*=0.908; *P*<0.001).

### Data analysis

All brief dream questions and full interviews were digitally recorded and transcribed for systematic content analysis conducted by two independent judges. Modified versions of the Subjective Experiences During Anesthesia (SEDA) and Orlinsky scales^28^, ^29^, ^30^ were used in the content analysis (Table 1) to (i) distinguish recall of specific content from no recall, (ii) distinguish disconnected experiences from connected experiences, and (iii) address the complexity and (iv) modality of experiences.

### Statistical methods

Statistical analyses were performed using SAS System version 9.4 for Windows (SAS Institute Inc., Cary, NC, USA). Inter-rater agreement of vigilance state scoring and content analysis were assessed with per cent agreement and Cohen's kappa (*Ҡ*) coefficient. Pearson's χ^2^ and Fisher's exact test (FET) were used to assess differences in rousability and prevalence and content of reports between anaesthetic agents (i.e. in between-subject comparisons). Logistic regression models with random intercept were used to assess the prevalence and content of reports between the different unresponsiveness periods during anaesthesia, between sleep stages, and between anaesthetic-induced unresponsiveness and sleep (i.e. in within-subject comparisons). Mann–Whitney *U*-test (*U*) was used in between-subject comparisons and linear mixed model with random intercept was used in within-subject comparisons, respectively, to assess the association between the measured drug plasma concentrations and rousability, recall rate of experiences, and type of recalled experiences. Logarithmic transformation was used in linear mixed models because of positively skewed distribution of concentration values. All tests were two-tailed, and an alpha level of 0.05 was considered statistically significant. For the main analyses, we also report the relative risk (RR) or the difference in prevalence with 95% confidence intervals (CIs).

## Results

### Awakenings and interviews

There were 76 successful awakenings and interviews obtained in Experiment 1 (Table 2). There was no difference in rousability for interviews between dexmedetomidine (16/20) and propofol (13/19) in UR1 (FET *P*=0.480; RR=1.17; 95% CI: 0.80–1.70), but drug plasma concentrations were lower in rousable compared with unrousable participants in UR1 for both anaesthetic agents (dexmedetomidine: *U*=69.0, *P*=0.007, *MedianRousable*=1.55 ng ml^−1^, and *MedianUnrousable*=3.51 ng ml^−1^; propofol: *U*=93.0, *P*=0.002, *MedianRousable*=1.20 μg ml^−1^, and *MedianUnrousable*=2.11 μg ml^−1^).

**Table 2:** Experiment 1: awakenings and interviews, and achieved states, with mean measured drug plasma concentrations for those participants who achieved the state. ∗Four dexmedetomidine and four propofol experiments were terminated before UR3. R, responsiveness; sd, standard deviation; UR, unresponsiveness.

	UR1, *N* of interviews/UR1, *N* of achieved states (%)	UR1, mean drug concentration (sd)	UR2, *N* of interviews/UR2, *N* of achieved states (%)	UR2, mean drug concentration (sd)	UR3, *N* of interviews/UR3, *N* of achieved states (%)	UR3, mean drug concentration (sd)	R3, mean drug concentration (sd)	All R periods, *N* of interviews/all UR periods, *N* of achieved states (%)
Both drugs	29/39 (74.4)		16/17 (94.1)		31/31 (100)			76/87 (87.4)
Dexmedetomidine	16/20 (80.0)	1.80 (0.84) ng ml^−1^	14/15 (93.3)	1.65 (0.38) ng ml^−1^	16/16∗(100)	3.27 (1.32) ng ml^−1^	1.64 (0.64) ng ml^−1^	46/51 (90.2)
Propofol	13/19 (68.4)	1.48 (0.60) μg ml^−1^	2/2 (100)	0.96 (0.20) μg ml^−1^	15/15∗(100)	2.46 (0.77) μg ml^−1^	1.11 (0.28) μg ml^−1^	30/36 (83.3)

In Experiment 1, we noted discrepancies between the participants' answers to the brief dream question and the full interview, but we used only the full interviews (with detailed content reports) in the analyses. Of note, five times (6.6%) the participants answered ‘yes’ to the brief dream question but in the full interview no longer recalled any content. Further, nine times (11.8%) the participants stated in the brief dream question that they were ‘unsure’ but reported dreaming in the full interview. Relatedly, 15 times (19.7%) the participants answered ‘no’ to the brief dream question but in the full interview reported experiences categorised as memory incorporation.

In Experiment 2, 36 out of the 37 participants fell asleep at least once but successful PET scans, and subsequent interviews were achieved with 32 participants. Notably, in Experiment 2, the same participant could be interviewed more than once from the same sleep stage.

### Experiences during anaesthetic-induced unresponsiveness and NREM sleep stages

In Experiment 1, 80.0% of dexmedetomidine-receiving and 73.7% of propofol-receiving participants recalled experiences (i.e. produced content reports) at least once (difference 6.3%; 95% CI −20.1% – 32.8%). Of the 76 interviews, 69.7% included content from the unresponsive period (for details, see Table 3). In Experiment 1, 71.9% of the participants recalled experiences with specific content at least once, and 64.4% of the 73 awakenings led to recall of specific content (Table 3).

**Table 3:** Prevalence of recalling experiences from anaesthetic-induced unresponsiveness (Experiment 1) and non-rapid eye movement sleep (Experiment 2). ∗Differences were not observed in drug plasma concentrations measured immediately before the interview (UR1, UR2, and R3 states combined) between those who reported no recall or content reports in either dexmedetomidine (*N*=39, *F*(1, 19)=0.384, *P*=0.543; *geometric mean ContentReport*=1.54 ng ml^−1^; *geometric mean NoRecall*=1.45 ng ml^−1^) or propofol (*N*=29, *F*(1, 10)=0.589, *P*=0.460; *geometric mean ContentReport*=1.05 μg ml^−1^; *geometric mean NoRecall*=1.18 μg ml^−1^) groups. CI, confidence interval; UR, unresponsiveness.

	Anaesthetic-induced unresponsiveness				Sleep				Difference in prevalence between sleep and anaesthesia (95% CI)
	Prevalence of report types (*n*)/number of achieved states (*N)* (% of recall)								
	*n* of report type/UR1 (%)	*n* of report type/UR2 (%)	*n* of report type/UR3 (%)	All UR periods combined∗ (%)	*n* of report type/N1 (%)	*n* of report type/N2 (%)	*n* of report type/N3 (%)	All sleep stages combined (%)	
No recall	6/29 (20.7)	4/16 (25.0)	6/31 (19.4)	16/76 (21.1)	4/11 (36.4)	12/33 (36.4)	5/29 (17.2)	21/73 (28.8)	−7.7% (−21.6% – 6.1%)
Dexmedetomidine	2/16 (12.5)	4/14 (28.6)	2/16 (12.5)	8/46 (17.4)					
Propofol	4/13 (30.7)	0/2 (0.0)	4/15 (26.7)	8/30 (26.7)					
White report	1/29 (3.4)	3/16 (18.7)	3/31 (9.7)	7/76 (9.2)	0/11 (0.0)	4/33 (12.1)	1/29 (3.5)	5/73 (6.8)	2.4% (−6.4% – 11.1%)
Dexmedetomidine	1/16 (6.3)	3/14 (21.4)	3/16 (18.8)	7/46 (15.2)					
Propofol	0/13 (0.0)	0/2 (0.0)	0/15 (0.0)	0/30 (0.0)					
Content report	22/29 (75.9)	9/16 (56.3)	22/31 (70.9)	53/76 (69.7)	7/11 (63.6)	17/33 (51.5)	23/29 (79.3)	47/73 (64.4)	5.4% (−9.7% – 20.4%)
Dexmedetomidine	13/16 (81.3)	7/14 (50.0)	11/16 (68.8)	31/46 (67.4)					
Propofol	9/13 (69.2)	2/2 (100.0)	11/15 (73.3)	22/30 (73.3)					

Differences in recalling experiences were not observed between drugs in different unresponsiveness periods (UR1 FET *P*=0.667; UR2 FET *P*=0.475; UR3 FET *P*=1.000) or within drug groups between unresponsiveness periods (dexmedetomidine *N*=46, *F*(2, 43)=1.720, *P*=0.191; propofol *N*=28, *F*(1, 26)=0.107, *P*=0.747). Note that with propofol, UR2 was excluded from between unresponsiveness period comparison because all observations belonged in the *Content report* category. The measured drug plasma concentrations did not associate with recall of experiences in either drug group (for statistical details, see Table 3 title). Differences between sleep stages N1, N2, and N3 in recalling experiences were not observed (*N*=73, *F*(2, 70)=2.158, *P*=0.123). When both drugs and all unresponsiveness periods and all sleep stages were combined in analysis, anaesthetic-induced unresponsiveness and NREM sleep did not differ in the prevalence of recalling experiences (i.e. in producing content reports) (*N*=149, *F*(1, 147)=0.306, *P*=0.581).

### Disconnected and connected experiences during anaesthetic-induced unresponsiveness and NREM sleep stages

Disconnected experiences (i.e. dreaming and memory incorporation of the research setting) were frequently reported, whilst recall of connected experiences (i.e. awareness) was rare (Table 4). Differences were not observed between drugs in any unresponsiveness periods in either the prevalence of dreaming (UR1 FET *P*=1.000; UR2 FET *P*=0.444; UR3 FET *P*=0.149) or memory incorporation (UR1 FET *P*=1.000; UR2 FET *P*=1.000; UR3 FET *P*=1.000). Similarly, no differences emerged within drug groups between unresponsiveness periods in dreaming (dexmedetomidine *N*=31, *F*(2, 28)=0.075, *P*=0.928; propofol *N*=20, *F*(1, 18)=2.790, *P*=0.112) or memory incorporation (dexmedetomidine *N*=31, *F*(2, 28)=0.082, *P*=0.922; propofol FET *P*=0.333). With propofol, UR2 was excluded from between unresponsiveness period comparison because all observations belonged in the *Dreaming* category. With propofol, in UR2 and UR3, the random intercept model could not be used because all observations belonged in the *MemoryIncorporation* category. For the associations between measured drug plasma concentrations and dreaming or memory incorporation, see Table 4 title. Lastly, the prevalence of dreaming (*N*=47, *F*(2, 44)=1.310, *P*=0.280) or memory incorporation (*N*=47, *F*(2, 44)=0.704, *P*=0.500) did not differ between sleep stages N1, N2, and N3. With all unresponsiveness periods and both drugs combined, anaesthetic-induced content reports included dreaming (*N*=100, *F*(1, 98)=0.662, *P*=0.418) and memory incorporation (*N*=100, *F*(1, 98)=1.635, *P*=0.204) as often as reports collected after NREM sleep awakenings (all sleep stages combined).

**Table 4:** Disconnected and connected experiences during anaesthetic-induced unresponsiveness (Experiment 1) and non-rapid eye movement sleep (Experiment 2). ∗Differences were not observed in drug plasma concentrations measured immediately before the interview (UR1, UR2, and R3 states combined) in the dexmedetomidine group between those who reported or did not report dreaming or memory incorporation (dreaming *N*=31, *F*(1, 14)=0.547, *P*=0.472, *geometric mean Dreaming*=1.58 ng ml^−1^, and *geometric mean NoDreaming*=1.48 ng ml^−1^; memory incorporation *N*=31, *F*(1, 14)=0.053, *P*=0.822, *geometric mean MemoryIncorporation*=1.55 ng ml^−1^, and *geometric mean NoMemoryIncorporation*=1.49 ng ml^−1^). In the propofol group, differences were observed in drug plasma concentrations measured immediately before the interview (UR1, UR2, and R3 states combined) between those who reported and did not report dreaming (*N*=21, *F*(1, 7)=10.698, *P*=0.014; *geometric mean Dreaming*=1.12 μg ml^−1^; *geometric mean NoDreaming*=0.83 μg ml^−1^). *Post hoc* analyses revealed that UR1 concentration was higher in those participants who reported dreaming (UR1 *U*=11.0; *P*=0.024; *MedianDreaming*=1.20 μg ml^−1^; *MedianNoDreaming*=0.79 μg ml^−1^). Statistically significant differences were not observed in measured UR1 drug plasma concentrations in the propofol group between those who reported and did not report memory incorporation (*U*=14.5; *P*=0.250; *MedianMemoryIncorporation*=0.83 μg ml^−1^; *MedianNoMemoryIncorporation*=1.21 μg ml^−1^) (other comparisons not applicable because of lack of observations in *NoMemoryIncorporation* category in UR2 and UR3). CI, confidence interval; UR, unresponsiveness.

	Anaesthetic-induced unresponsiveness				Sleep				
	Prevalence of recalled content (*n*)/number of content reports (*N*) (% of recall)								Difference in prevalence between sleep and anaesthesia (95% CI)
	UR1	UR2	UR3	All UR periods combined∗	N1	N2	N3	All sleep stages combined	
Disconnected experiences									
Dreaming	12/22 (54.5)	5/9 (55.6)	16/22 (72.7)	33/53 (62.3)	6/7 (85.7)	6/17 (35.3)	12/23 (52.2)	24/47 (51.1)	11.2% (−8.2% – 30.6%)
Dexmedetomidine	7/13 (53.8)	3/7 (42.9)	6/11 (54.5)	16/31 (51.6)					
Propofol	5/9 (55.6)	2/2 (100.0)	10/11 (90.9)	17/22 (77.3)					
Memory incorporation	18/22 (81.8)	8/9 (88.9)	21/22 (95.5)	47/53 (88.7)	4/7 (57.1)	14/17 (82.4)	19/23 (82.6)	37/47 (78.7)	10.0% (−4.5% – 24.4%)
Dexmedetomidine	11/13 (84.6)	6/7 (85.7)	10/11 (90.9)	27/31 (87.1)					
Propofol	7/9 (77.8)	2/2 (100.0)	11/11 (100.0)	20/22 (90.9)					
Connected experiences									
Awareness	0/22 (0.0)	2/9 (22.2)	1/22 (4.5)	3/53 (5.7)	1/7 (14.3)	1/17 (5.9)	1/23 (4.3)	3/47 (6.4)	-0.7% (−10.1% – 8.6%)
Dexmedetomidine	0/13 (0.0)	1/7 (14.3)	0/11 (0.0)	1/31 (3.2)					
Propofol	0/9 (0.0)	1/2 (50.0)	1/11 (9.1)	2/22 (9.1)					

### Complexity and modality of experiences during anaesthetic-induced unresponsiveness and NREM sleep stages

In Experiment 1, there were no differences between drugs (UR1 FET *P*=0.303; UR2 FET *P*=1.000; UR3 FET *P*=1.000) or within drugs between different unresponsiveness periods in the complexity of dreaming (i.e. whether the dream experience was non-perceptual, static, or dynamic) (dexmedetomidine *N*=31, *F*(1, 28)=0.075, *P*=0.928; propofol *N*=20, *F*(1, 18)=2.790, *P*=0.112). Similarly, complexity of memory incorporation experiences did not differ between drugs (UR1 FET *P*=0.353; UR2, not applicable because of small *n*; UR3 FET *P*=0.796) or within drugs between unresponsiveness periods (dexmedetomidine *N*=31, *F*(2, 28)=0.082, *P*=0.922; propofol FET *P*=0.333). With propofol, UR2 was excluded from between unresponsiveness period comparison because of lack of observations. Finally, sleep stages did not differ with respect to complexity of dreaming (*N*=24, *F*(2, 21)=2.929, *P*=0.076) or memory incorporation. Comparison of the complexity of memory incorporation between sleep stages could not be assessed with the random intercept model because all observations belonged in the *StaticReport* category. In the pooled analysis, dreaming (FET *P*=0.020) and memory incorporation (FET *P*=0.015) were more often dynamic during anaesthetic-induced unresponsiveness than during NREM sleep (Table 5). Notably, there were only two differences in experiential modalities between anaesthetic-induced unresponsiveness and NREM sleep, but because of the low number of specific modalities, these results should be viewed as preliminary (for statistical details, see Table 5).

**Table 5:** Complexity and modality of experiences in reports from anaesthetic-induced unresponsiveness (Experiment 1) and non-rapid eye movement (NREM) sleep (Experiment 2). ∗Negative emotions were more often present during anaesthetic-induced unresponsiveness than during NREM sleep in reports that included dreaming (*N*=56, *F*(1, 54)=4.438, *P*=0.040). ∗∗Kinaesthetic/vestibular sensations (*N*=83, *F*(1, 81)=5.796, *P*=0.018) were more often experienced during anaesthetic-induced unresponsiveness than during NREM sleep in reports that included memory incorporation.

	Dreaming		Memory incorporation	
	Anaesthesia, N of reports that include dreaming = 33	Sleep, *N* of reports that include dreaming = 24	Anaesthesia, *N* of reports that include memory incorporation = 47	Sleep, *N* of reports that include memory incorporation = 37
	*N* of reports that include specific content (%)		*N* of reports that include specific content (%)	
Complexity				
No perceptual content	3 (9.1)	0 (0)	3 (6.4)	0 (0)
Static report	9 (27.3)	15 (62.5)	37 (78.7)	37 (100)
Dynamic report	21 (63.6)	9 (37.5)	7 (14.9)	0 (0)
Modality				
Sensory–perceptual				
Visual	30 (90.1)	20 (83.3)	5 (10.6)	7 (18.9)
Auditory	15 (45.5)	10 (41.7)	37 (78.7)	29 (78.4)
Gustatory	1 (3.0)	0 (0)	0 (0)	0 (0)
Olfactory	0 (0)	0 (0)	0 (0)	0 (0)
Interoceptive	2 (6.0)	0 (0)	2 (4.3)	2 (5.4)
Kinaesthetic/vestibular	13 (39.4)	6 (25.0)	17 (36.2)∗∗	5 (13.5)∗∗
Tactile	1 (3.0)	0 (0)	14 (29.8)	14 (37.8)
Nociceptive and thermoceptive	1 (3.0)	0 (0)	7 (14.9)	6 (16.2)
Affective states				
Positive	13 (39.4)	7 (29.2)	5 (10.6)	2 (5.4)
Negative	11 (33.3)∗	1 (4.2)∗	3 (6.4)	0 (0)
Cognition	15 (45.5)	6 (25.0)	12 (25.5)	6 (16.2)
Out-of-body experience	0 (0)	0 (0)	0 (0)	0 (0)
Sense of presence	2 (6.0)	0 (0)	8 (17.0)	1 (2.7)

## Discussion

We compared recall and content of subjective experiences reported after awakenings from anaesthetic-induced unresponsiveness and NREM sleep in the same male participants. As hypothesised, we found no significant differences in the recall rate or main content of experiences between anaesthesia and natural sleep, although disconnected experiences during anaesthesia tended to be more often dynamic than experiences during sleep. Our results are, in general, in line with previous findings regarding experiences in experimental anaesthesia and NREM sleep.^15^^,^^28^, ^29^, ^30^, ^31^, ^32^, ^33^ The phenomenal level of similarity in the recall rate and content of experiences during dexmedetomidine- or propofol-induced unresponsiveness and NREM sleep is compatible with the mechanistic and neurophysiological similarities of these states.

Rousability did not differ between dexmedetomidine and propofol, but lower drug plasma concentrations were related to rousability with both drugs, as expected. Experiences with specific remembered content were reported in about two-thirds of the awakenings, and the reported experiences almost always reflected disconnected contents of consciousness. The recall of experiences after unresponsive periods (no recall, white report, or content report) or the type of recalled content (dreaming or memory incorporation) did not differ between anaesthetic agents or sleep stages N1, N2, and N3, but in the propofol group those who reported dreaming after waking up from the first unresponsive period had higher measured drug plasma concentrations than those who did not report dreaming. Memory incorporation, that is, contents that reflected some remembered aspects of the research environment but not direct awareness of it, was present in more than 80% of the reports in all awakenings (except for the N1 sleep stage). Interestingly, memory incorporation experiences were not related to drug plasma concentrations with either dexmedetomidine or propofol, even though a higher probability of sensory intrusions from the research environment could have been expected to be related to lower concentrations.

Notably, in 50.9% of anaesthesia reports and 29.8% of sleep reports, dream-like content was present in the same report as memory incorporation content, that is, content related and unrelated to the research setting co-existed in the same report. Similarly, all six reports that included awareness aslo included memory incorporation and all but one dreaming. These findings not only add evidence that disconnected conscious experiences persist and are prevalent in unresponsive states, but they show that when probed with a detailed interview, experiences reflecting different degrees of connectedness can interweave. Experiences during both anaesthesia and sleep often superimposed **hallucinatory** dream-like and *realistic* memory incorporation elements (e.g. ‘**My girlfriend was sitting next to me** *on this bed in this scanner room*, and **we were discussing holiday plans**’), whereas other reports depicted events that had actually happened either before or during unresponsiveness (e.g. ‘There were *people in the next room, I heard them moving chairs,* and *at some point, somebody touched my arm*’). We can only speculate whether these incorporations reflect episodic memories of events that had already taken place before unresponsiveness ensued, similar to how dreams collected in an experimental setting frequently reflect sleep laboratory elements,^36^ or whether in some cases the incorporations could signify connected consciousness, similar to how dreams can, directly or in an associative manner, reflect stimulus incorporation into contents of consciousness during sleep.^37^ Although we have conceptualised memory incorporation as disconnected contents of consciousness, it is possible that these experiences mark continuing integration of stimuli from both external and internal sources, from wakefulness into unresponsiveness. Our evidence suggests that the line between sensory connectedness and disconnectedness is not absolute but gradual, including a grey zone where internally and externally generated experiences become entangled with each other and with memories. This might reflect the multisensory integration of subjective experiences that normally produces the unity of conscious perception.

Given that we cannot exclude the possibility that connected phenomenal consciousness was present in some of those occasions when memory incorporation was reported, our findings might bear some relevance for clinical practices. Administration of anaesthetics in the current study corresponds to mild-to-moderate sedation rather than surgical anaesthesia and both responsive and unresponsive sedation are widely used in clinical contexts. When unresponsive sedation is used with the hope of abolishing connectedness, it might be important to address retrospective recall to assess whether this goal was successfully achieved. In previous studies on dreaming during surgical anaesthesia, the contents of dreams have mostly been related to everyday life or the surgical setting and operating room.^20^^,^^25^ Distinguishing whether experiences that reflect the surgical setting originate purely from episodic memory sources or might occasionally reflect the processing of external stimuli on a phenomenal level during the anaesthetic procedure deserves further attention, given that the amnesic effects of anaesthetics tend to abolish recall of even behavioural indications of connected experiences.^38^ It likely is much more difficult to draw the line between disconnected memory incorporation and connected stimulus incorporation in an experimental than in a surgical setting, where nociceptive stimuli are typically present.

Although the amnesic effects of anaesthetics are well documented,^38^ natural sleep also induces retrograde amnesia.^39^ Therefore, distinguishing unconsciousness from amnesia remains a challenge both in anaesthesia and sleep studies. In our study, whilst awakenings without recall of experiences might be interpreted to reflect unconsciousness, reports lacking recall and especially white reports, which are often interpreted to reflect forgotten experiences, only show that the preceding unresponsive state was associated with amnesia, not that it was characterised by unconsciousness.

The strengths of the present study lie in the direct comparison of experiences in two different unresponsive states utilising the same methods in a within-subject design, thus mitigating the effect of individual trait differences (such as retrospective memory for subjective experiences) on outcomes.^21^^,^^40^ Further, the detailed interview and the multi-staged categorisation of experiences are key strengths of the study. The current experimental design with systematic assessment of participants' experiential states during anaesthesia and sleep contributes a methodological insight that could be used in future studies. Studying connectedness and disconnectedness induced with various means in the same subjects and utilising various measurement tools could help resolve both empirical and theoretical issues in the science of consciousness.

The current study also suffers from several limitations. First, sample size was limited. However, the reported 95% CIs exclude large differences between the drug groups or between anaesthesia and sleep and suggest that our sample size was adequate to test our hypotheses. Second, our sample included only males because of the potential risks of exposing fertile females to radioactive tracers. We are unable to exclude potential sex differences on the outcomes, and future investigations on this topic are needed.

As to other weaknesses in design, in Experiment 1 the full interviews were slightly delayed because of intervening PET scans. Delays might have accentuated the amnesic effects of anaesthetics and distorted the reports. To reduce memory bias, we presented the brief dream question immediately after arousal and conducted the full interview on average 7.5 min later. This, however, led to discrepancies in participants' answers. On a few occasions, participants answered ‘yes’ to the brief dream question (i.e. they recalled a dream) but in the full interview no longer recalled any content, which likely reflects the amnesic effects of the anaesthetics. On other occasions, participants were ‘unsure’ in the brief dream question but then reported dreaming in the full interview. Possibly, when participants had time to think about the experience during the PET scan, they recalled details that could not be immediately reported, that is, an immediate white report transformed into a content report. Another explanation might be that despite responsiveness tests that verified responsiveness before and after the PET scan, participants' state of consciousness fluctuated, and between the brief dream question and the full interview their contents of consciousness alternated between disconnected and connected experiences. Participants might have had brief episodes of internally generated thoughts and imagery during this responsive period, similar to mind wandering or sleep onset hypnagogia, and thus in the full interview they reported hallucinatory dream-like experiences that they had during the scan just before the full interview.

The largest discrepancy was observed between answering ‘no’ to the brief dream question and reporting memory incorporation in the full interview. This discordance could point to the possibility that some memory incorporations occurred during the responsive period, between the brief dream question and full interview. Yet, memory incorporation experiences were not related to drug plasma concentrations, that is, lower concentrations did not lead to higher levels of connectedness and incorporation from the responsive period. Another possibility is that directly inquiring about the incorporation of the research environment in the interview might have led to over-representation of such content. However, memory incorporation rate in the present study is not substantially higher than in our previous study utilising immediate interviews,^29^ suggesting that the short responsive period before the full interview or specific questions tapping into incorporated experiences do not alone account for the high levels of memory incorporation. Finally, participants might have interpreted the brief dream question to refer to similar types of hallucinated content as typical dreams. Thus, they truthfully answered ‘no’ to recalling dreams, but omitted incorporation experiences, reporting them only in the full interview. This highlights the potential effects of ambiguous interview questions or concepts used by researchers.

For future studies, we stress the importance of clarifying concepts to participants, asking unambiguous questions, and conducting immediate interviews to diminish the time lag between the experience and the report, and to mitigate amnesic effects, memory bias, and filling in the gaps by confabulation. We support use of thorough and transparently reported interview and classification procedures and clearly defined categories in research of anaesthesia-related experiences.

### Conclusions

Our findings support the hypothesis that anaesthetic-induced unresponsiveness and NREM sleep are both characterised by disconnected conscious experiences with highly similar recall frequencies and content. These findings align with the mechanistic and neurophysiological similarities between anaesthetic-induced unresponsiveness and NREM sleep. Although anaesthetic-induced unresponsiveness in an experimental setting is not comparable with surgical anaesthesia and rather corresponds to moderate sedation, our results have relevance for clinical practices, both for sedation and surgical anaesthesia. We speculate that in some cases surgical anaesthesia does not represent a state of unconsciousness, but that patients disconnected from the environment might experience internally generated experiences similar to those experienced under NREM sleep. This poses no problem for successful surgical anaesthesia as long as patients remain disconnected from and unaware of external and bodily stimuli.

## Authors’ contributions

Principal investigators: KV, HS

Study conception/design: KV, REK, AS, JL, KK, AR, HS

Study conduct: KV, LR, REK, AS, JL, KK, JK

Data analysis: KV, LR, REK, JK

Statistical analyses: TV, KV

Writing of paper: KV, LR, REK

Revising of paper: all authors

## Declaration of interest

The authors declare no conflicting interests.

## Funding

10.13039/501100002341Academy of Finland, Helsinki, Finland (266467 and 266434); Jane and Aatos Erkko Foundation, Helsinki, Finland; VSSHP-EVO (13323 and L3824); Doctoral Program of Clinical Investigation, University of Turku Graduate School, Turku, Finland to LR and AS; The Paulo Foundation, Espoo, Finland to AS; The Finnish Medical Foundation, Helsinki, Finland to AS; The Orion Research Foundation, Espoo, Finland to AS; Signe and Ane Gyllenberg Foundation, Helsinki, Finland to KV.
